# ARIH1 Inhibition Promotes Microtubule Stability and Sensitizes Breast Cancer Cells to Microtubule-Stabilizing Agents

**DOI:** 10.3390/cancers17050782

**Published:** 2025-02-25

**Authors:** Mohamed Elshaer, Breege V. Howley, Philip H. Howe

**Affiliations:** 1 Department of Biochemistry and Molecular Biology, Medical University of South Carolina, Charleston, SC 29425, USA; 2 Labeled Compounds Department, Hot Labs Center, Egyptian Atomic Energy Authority, Cairo 13759, Egypt; 3 Hollings Cancer Center, Medical University of South Carolina, Charleston, SC 29425, USA

**Keywords:** MAP4, ARIH1, chemotherapy, microtubules stability, chemoresistance, biomarker, paclitaxel

## Abstract

**Background:** Microtubule dynamics play a pivotal role in cancer progression and response to chemotherapeutics. Identifying regulators of microtubule stability can provide new therapeutic targets and predictive biomarkers for cancer treatment. **Methods:** We investigated the role of ARIH1, an E3 ubiquitin ligase, in breast cancer by analyzing clinical datasets to assess its expression levels and prognostic significance. Functional studies were conducted in breast cancer cell lines to evaluate the impact of ARIH1 depletion on microtubule stability, MAP4 regulation, and paclitaxel sensitivity. **Results:** Clinical dataset analysis revealed that ARIH1 expression is significantly elevated in breast cancer tissues and correlates with poor prognosis and reduced recurrence-free survival. High ARIH1 expression stratifies patients into high-risk groups, underscoring its potential as a prognostic biomarker. Functional studies demonstrated that ARIH1 loss led to upregulation of MAP4, a microtubule-associated protein, resulting in microtubule stabilization via increased tubulin acetylation and enhanced spindle organization. This stabilization sensitized breast cancer cells to paclitaxel treatment, leading to reduced cell viability, impaired colony formation, and increased apoptosis in ARIH1-deficient cells. **Conclusions:** Our findings identify ARIH1 as a novel regulator of microtubule dynamics in breast cancer. ARIH1 suppression enhances paclitaxel sensitivity, highlighting its potential as both a therapeutic target and a biomarker for predicting treatment response and patient outcomes in breast cancer.

## Simple Summary

Chemotherapy drugs like paclitaxel are widely used to treat breast cancer by stabilizing microtubules, structures essential for cell division. However, resistance to paclitaxel limits its effectiveness in many patients. In this study, we identify ARIH1, a protein that regulates microtubule stability, as a potential therapeutic target and biomarker in breast cancer. We found that high ARIH1 expression is associated with poor patient outcomes, and its loss leads to an increase in MAP4, a microtubule-associated protein that enhances microtubule stability. As a result, breast cancer cells with reduced ARIH1 levels become more sensitive to paclitaxel, leading to greater cancer cell death. These findings suggest that ARIH1 could be used to predict treatment response and may be a target for new therapies aimed at overcoming chemotherapy resistance. By targeting ARIH1, we may improve the effectiveness of microtubule-stabilizing drugs in breast cancer treatment.

## 1. Introduction

Breast cancer remains the most prevalent cancer among women worldwide and is a leading cause of cancer-related mortality [1,2]. While advancements in targeted therapies and chemotherapeutics have improved outcomes, resistance to standard treatments, such as microtubule-targeting agents (MTAs) like paclitaxel, poses a significant clinical challenge [3,4]. Paclitaxel exerts its cytotoxic effects by stabilizing microtubules, arresting cells in mitosis, and inducing apoptosis. However, resistance to paclitaxel, driven by alterations in microtubule dynamics and associated regulatory proteins, frequently limits its therapeutic efficacy [5,6].

Microtubule-associated proteins (MAPs) play a critical role in maintaining microtubule stability and dynamics, which are key determinants of cellular response to MTAs. Among these, MAP4 is a well-documented stabilizer of microtubules that enhances tubulin acetylation and reduces microtubule depolymerization, processes critical for microtubule-targeting drug sensitivity [7,8]. Overexpression of MAP4 has been shown to sensitize cancer cells to paclitaxel by promoting microtubule stabilization, demonstrating the importance of the pre-cellular status of microtubules in determining their response to microtubule-stabilizing agents [9,10,11,12,13,14,15,16,17]. Despite the importance of MAP4 in microtubule dynamics, the upstream regulators that modulate its activity remain poorly understood.

ARIH1, an E3 ubiquitin ligase, has emerged as a multifunctional protein involved in various cellular processes, including protein degradation, mitophagy, and stress response pathways [18,19,20,21,22]. Recent studies have linked ARIH1 to cancer progression and chemoresistance, particularly in breast cancer and other solid tumors [23]. However, its specific role in microtubule dynamics and MTA resistance has not been explored. Clinical datasets suggest that ARIH1 is frequently overexpressed in breast cancer and associated with poor patient outcomes, making it a promising candidate for therapeutic targeting and biomarker development.

In this study, we investigated the role of ARIH1 in regulating microtubule dynamics and its impact on breast cancer cell response to paclitaxel. We identified MAP4 as a downstream effector of ARIH1, with ARIH1 depletion leading to MAP4 upregulation and enhanced microtubule stabilization. These stabilized microtubules sensitized ARIH1-deficient cells to paclitaxel, resulting in reduced cell viability, impaired colony formation, and increased apoptosis. Furthermore, we demonstrated that high ARIH1 expression stratifies breast cancer patients into high-risk groups with poor survival outcomes, highlighting its potential as a prognostic biomarker.

Our findings uncover a novel role for the ARIH1-MAP4 axis in microtubule regulation and establish ARIH1 as a dual-purpose therapeutic target and biomarker for breast cancer treatment. This study provides a strong rationale for targeting ARIH1 to overcome paclitaxel resistance and improve treatment outcomes in breast cancer.

## 2. Methods

### 2.1. Cell Culture

PY8119 (CRL-3278) breast cancer cell lines were obtained from ATCC. SUM159 cell lines were kindly provided by Dr. Ethier (Medical University of South Carolina, Charleston, SC, USA). Cells were cultured in Dulbecco’s Modified Eagle Medium (DMEM) supplemented with 10% fetal bovine serum (FBS), 100 U/mL penicillin, and 100 µg/mL streptomycin (Gibco, Grand Island, NE, USA) at 37 °C in a humidified atmosphere of 5% CO_2_. The cells were routinely tested for mycoplasma contamination.

### 2.2. ARIH1 Perturbation

ARIH1 was knocked-down in SUM159 cells using ARIH1-targeting shRNA and scrambled control shRNA constructs. Stable cell lines were generated using lentiviral transduction, and successful knockdown was validated by Western blot analysis, qPCR, and immunofluorescence. ARIH1 was knocked out in PY8119 cells using ARIH1 targeting CRISPR Cas9 technology. ARIH1 KO cell lines were generated by electroporating the CRISPR Cas9 system components into the cells, and successful knockout was validated by Western blot.

#### 2.2.1. Clonogenic Assay

To evaluate the impact of ARIH1 perturbation on the long-term proliferative capacity of breast cancer cells, clonogenic assays were performed. PY8119 (WT and ARIH1 KO) and SUM159 (scrambled control and ARIH1 KD1) cells were seeded at a low density (500 cells per well) in 6-well plates. After 24 h, the cells were treated with paclitaxel for 10 days. After the treatment period, the colonies were fixed with methanol, stained with 0.5% crystal violet, washed, and air-dried.

#### 2.2.2. Flow Cytometry for Cell Cycle Analysis

To assess cell cycle distribution and apoptosis, flow cytometry analysis was performed using 7-Aminoactinomycin D (7-AAD) staining. PY8119 (WT and ARIH1 KO) cells were treated with paclitaxel (50 nM, 100 nM, and 200 nM) for 48 h. After treatment, cells were harvested, washed twice with cold phosphate-buffered saline (PBS), fixed in cold 70% ethanol for at least 30 min at 4 °C, and stained with 25 µg/mL 7-AAD in PBS. Samples were incubated for 30 min at room temperature in the dark before analysis. Flow cytometry was performed using a BD LSRFortessa™ flow cytometer. At least 15,000 events per sample were recorded. Data were analyzed using FlowJo software to quantify sub-G1 (apoptotic cells) and G2/M peaks (cell cycle arrest).

#### 2.2.3. Western Blot Analysis

Cells were lysed in RIPA buffer containing protease and phosphatase inhibitors (Roche). Protein concentrations were determined using a BCA assay (Pierce). Equal amounts of protein (20–30 µg) were separated by SDS-PAGE and transferred to PVDF membranes. Membranes were blocked with 5% non-fat milk in TBST for 1 h and incubated overnight with primary antibodies against ARIH1 (14949-1-AP; proteintech), MAP4 (11229-1-AP; proteintech), acetylated tubulin (66200-1-Ig; proteintech), and HSP90 (sc13119: Santa Cruz Biotechnology). After washing, the membranes were incubated with HRP-conjugated secondary antibodies for 1 h. Protein bands were visualized using the BioRad ChemiDoc system.

#### 2.2.4. Immunofluorescence

Cells grown on coverslips were fixed with 4% paraformaldehyde for 15 min, permeabilized with 0.1% Triton X-100, and blocked with 5% bovine serum albumin (BSA) for 30 min. Coverslips were incubated with primary antibodies against beta-tubulin, MAP4, or acetylated tubulin overnight at 4 °C. After washing with PBS, coverslips were incubated with Alexa Fluor-conjugated secondary antibodies (Invitrogen) for 1 h at room temperature. Nuclei were stained with DAPI (1 µg/mL) for 5 min. Images were acquired using an Olympus FV10i laser-scanning confocal microscope, and an analysis was performed using Fiji software 2.9.0.

#### 2.2.5. Drug Sensitivity Assays and IC50 Calculation

To determine the sensitivity of breast cancer cells to paclitaxel, CellTiter-Glo^®^ Luminescent Cell Viability Assays were performed, and the half-maximal inhibitory concentration (IC50) was calculated. PY8119 (WT and ARIH1 KO) and SUM159 (scrambled control and ARIH1 KD1/KD2) cells were plated in 96-well plates at a density of 5000 cells per well in triplicate. After 24 h of seeding, the cells were treated with paclitaxel at concentrations ranging from 0 to 200 nM for 96 h.

At the end of the treatment period, 100 µL of CellTiter-Glo^®^ reagent were added to each well according to the manufacturer’s protocol. Plates were incubated at room temperature for 10 min, and luminescence was measured using a microplate reader. Cell viability was expressed as a percentage relative to untreated control wells.

IC50 values were determined by fitting dose–response curves to a four-parameter logistic model using GraphPad Prism software 10.4.1. Data were expressed as mean ± standard deviation from three independent experiments. Fold changes in IC50 were calculated to compare ARIH1-perturbed cells to controls.

#### 2.2.6. qPCR Analysis

Total RNA was extracted from cell lines using a Zymo research RNA extraction kit. cDNA synthesis was performed using qScript cDNA synthesis kits with 100–1000 ng of total RNA (Quantabio, Beverly, MA, USA). Real-time quantitative PCR was conducted using iQ SYBR Green Supermix (Bio-Rad, Hercules, CA, USA) using CFX384 Real-Time System (BioRad). Relative gene expression was calculated using RFX Manager software, and genes were normalized to GAPDH internal control.

#### 2.2.7. Statistical Analysis

All experiments were performed in triplicate unless otherwise stated. Data are presented as mean ± standard deviation (SD). Statistical significance was determined using unpaired two-tailed Student’s *t*-tests or one-way ANOVA with Tukey’s post hoc test, as appropriate. *p*-values < 0.05 were considered statistically significant. Data analysis was performed using GraphPad Prism version 10.4.1.

## 3. Results

### 3.1. ARIH1 Is a Potential Target for Breast Cancer Therapy

**Figure 1:**
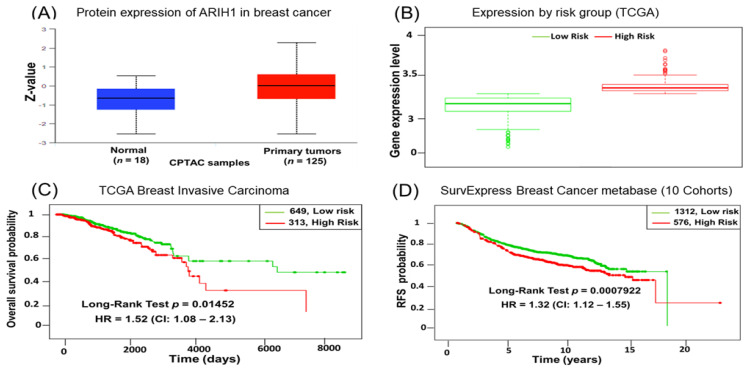
Elevated expression of ARIH1 is associated with high-risk breast cancer and poor survival outcomes. (**A**) Boxplot showing ARIH1 protein expression levels in normal (*n* = 18) versus primary breast tumor samples (*n* = 125) from CPTAC datasets. Z-values indicate significantly higher ARIH1 expression in primary tumors compared to normal tissue. (**B**) ARIH1 gene expression levels in high-risk versus low-risk breast cancer groups using TCGA data. Boxplots reveal elevated ARIH1 expression in the high-risk group. (**C**) Kaplan–Meier survival analysis indicating overall survival probability in low-risk (*n* = 649) and high-risk (*n* = 313) breast cancer patients. Patients with high ARIH1 expression exhibit significantly reduced overall survival (log-rank test, *p* = 0.01452; HR = 1.52, 95% CI: 1.08–2.13). (**D**) Kaplan–Meier analysis of recurrence-free survival (RFS) in low-risk (*n* = 1312) and high-risk (*n* = 576) groups. High ARIH1 expression correlates with poorer recurrence-free survival (log-rank test, *p* = 0.0007922; HR = 1.32, 95% CI: 1.12–1.55).

We first explored the role of ARIH1 in breast cancer by examining its expression in breast cancer tissue compared to normal breast tissue. An analysis of the CPTAC dataset revealed significantly higher ARIH1 protein levels in primary breast tumors (*n* = 125) compared to normal tissues (*n* = 18) (Figure 1A). These findings suggest that ARIH1 is upregulated in breast cancer and may play a role in tumor progression. Then, breast cancer patients from the TCGA and SurvExpress databases were stratified into high-risk and low-risk groups based on their risk scores, which are a weighted linear combination of gene expression values, where the weights are derived from Cox proportional hazards regression [24,25]. Further, we evaluated ARIH1 expression in high-risk and low-risk breast cancer groups. ARIH1 mRNA expression was significantly elevated in the high-risk group compared to the low-risk group, further supporting its association with aggressive breast cancer subtypes (Figure 1B). We assessed the prognostic value of ARIH1 by performing Kaplan–Meier survival analyses. Using TCGA data (*n* = 962), the patients were stratified into high-risk (*n* = 313) and low-risk (*n* = 649) groups based on ARIH1 expression. Patients with high ARIH1 expression exhibited significantly poorer overall survival (OS) compared to those with low expression (log-rank test, *p* = 0.01452; HR = 1.52, 95% CI: 1.08–2.13) (Figure 1C). In addition, we validated these findings by analyzing recurrence-free survival (RFS) using data from 10 independent cohorts available in the SurvExpress database (*n* = 1888). Patients were divided into high-risk (*n* = 576) and low-risk (*n* = 1312) groups. High ARIH1 expression correlated with significantly reduced RFS (log-rank test, *p* = 0.0007922; HR = 1.32, 95% CI: 1.12–1.55) (Figure 1D). These results establish ARIH1 as a potential biomarker for breast cancer prognosis. Its elevated expression in high-risk groups and association with poor survival outcomes highlight its potential as a therapeutic target for breast cancer management.

### 3.2. ARIH1 Regulates MAP4, a Microtubule-Associated Protein, in Breast Cancer Cells

**Figure 2:**
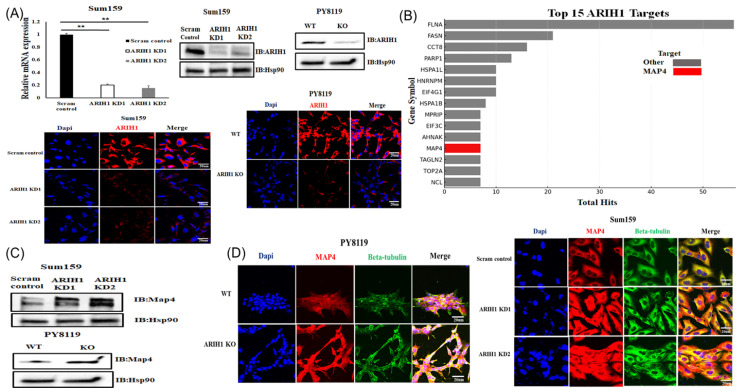
ARIH1 regulates MAP4 to modulate microtubule stability. (**A**) The efficiency of stable ARIH1 knockdown in SUM159 and ARIH1 knockout in PY8119 cells is shown by qPCR, Western blot, and immunofluorescence analysis of ARIH1. (**B**) Bar chart showing the top 15 interactors identified in the BioID experiment, ranked by total hits in biotin-treated samples. MAP4, highlighted in red, is among the top interactors identified. Other interactors are shown in gray. (**C**) Western blot analysis of MAP4 expression in PY8119 and Sum159 cells. Hsp90 was used as a loading control. (**D**) Immunofluorescence analysis of MAP4 (red), beta-tubulin (green), and nuclei stained with DAPI (blue) in PY8119 and Sum159 cells. Scram control is stable monoclonal control SUM159, generated by viral transduction of SUM159 cells with scrambled control shRNA construct. ARIH1 KD1 and KD2 are two different stable ARIH1 knockdown monoclones, generated by viral transduction of SUM159 by ARIH1 targeting shRNA construct. WT is wild-type PY8119 cells, generated by electroporating Cas9 enzyme alone into PY8119 cells. ARIH1 KO is ARIH1 knockout PY8119 cells, generated by electroporating the ARIH1 targeting CRISPR Cas9 system into PY8119 cells. Scale bar of 20 µM was used in immunofluorescence analysis. Error bars represent the mean ± standard deviation from three independent experiments. ** *p* < 0.01.

To explore the role of ARIH1 in cancer progression, we stably silenced ARIH1 in both SUM159 and Py8119 cells through shRNA KD and CRISPR Cas9 KO, respectively. Western blot, qPCR, and immunofluorescence analysis of ARIH1 confirmed efficient stable knockdown of ARIH1 in two different clones of SUM159 cells (ARIH1 KD1 and KD2) transduced with ARIH1-specific shRNA construct compared to scrambled controls (scram control) and efficient knockout of ARIH1 in PY8119 cells using CRISPR Cas9. (Figure 2A).

We utilized a BioID system that was previously established by our lab to identify ARIH1-interacting targets [20] and identified MAP4 as an ARIH1-interacting protein (Figure 2B). MAP4 is a microtubule-associated protein known to regulate microtubule stability [9,10,11]. Western blot analysis confirmed the differential expression of MAP4 in PY8119 and SUM159 cells under various conditions (Figure 2C). In SUM159 cells, MAP4 protein levels were markedly elevated in ARIH1 knockdown (KD1 and KD2) cells relative to scrambled controls (scram control) (Figure 2C, upper panel). In PY8119, MAP4 expression was similarly increased (Figure 2C, lower panel) in ARIH1 knockout (KO) cells compared to wild-type (WT) cells. These results suggest that ARIH1 negatively regulates MAP4 protein levels, either directly or indirectly.

Immunofluorescence analysis revealed a spatial relationship between MAP4 and beta-tubulin in PY8119 and SUM159 cells. In PY8119 WT cells, MAP4 exhibited a diffuse cytoplasmic distribution, with lower co-localization with beta-tubulin (Figure 2D, left panel). In contrast, ARIH1 KO cells displayed significantly enhanced MAP4 expression and co-localization with beta-tubulin, forming bundled microtubule structures. Similarly, in SUM159 cells, MAP4 expression and co-localization with beta-tubulin were significantly increased in ARIH1 KD1 and KD2 cells compared to scram control cells (Figure 2D, right panel). This was accompanied by the formation of prominent microtubule bundles. These findings suggest that ARIH1 deficiency leads to increased MAP4 expression and localization to microtubules.

### 3.3. ARIH1 Loss Promotes Microtubule Stabilization in Breast Cancer Cells

**Figure 3:**
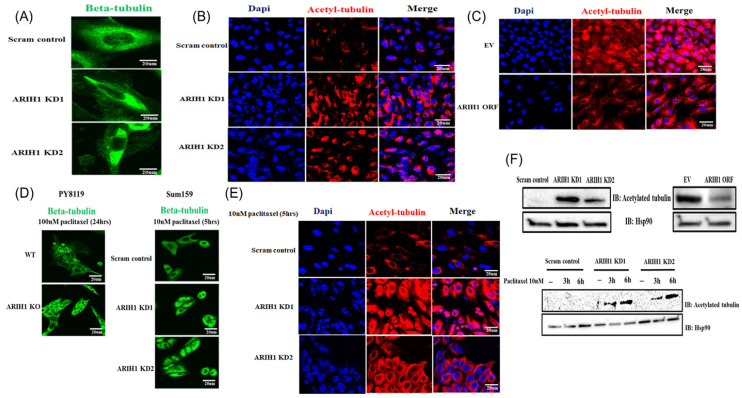
ARIH1 regulates microtubule stability. (**A**) Representative immunofluorescence images showing Beta-tubulin localization in SUM159 cells with scrambled control or ARIH1 knockdown. Cells were stained with Beta-tubulin (green) and imaged by confocal microscopy. (**B**) Immunofluorescence staining of acetylated tubulin (red) in SUM159 cells with scrambled control or ARIH1 knockdown. DAPI (blue) marks the nuclei, and merged images show the localization of acetylated tubulin. (**C**) SUM159 cells transduced with empty vector (EV) or ARIH1 overexpression (ARIH1 ORF) were stained for acetylated tubulin (red) and DAPI (blue). Merged images display reduced acetylation of tubulin upon ARIH1 overexpression. (**D**) Beta-tubulin organization in SUM159 and PY8119 cells treated with paclitaxel (10 nM for 5 h in SUM159 or 100 nM for 24 h in PY8119). Images show wild type (WT) or ARIH1 knockout (KO) in PY8119 cells and ARIH1 knockdown (KD1, KD2) in SUM159 cells. (**E**) Immunofluorescence staining for acetylated tubulin in SUM159 cells treated with 10 nM paclitaxel for 5 h under scrambled control or ARIH1 knockdown conditions (KD1, KD2). DAPI (blue) marks nuclei, and merged images highlight acetylated tubulin distribution. (**F**) Western blot analysis of acetylated tubulin levels in SUM159 cells. The left panel shows acetylated tubulin levels in scrambled control and ARIH1 knockdown (KD1, KD2) cells. The right panel shows acetylated tubulin levels in cells transduced with empty vector (EV) or ARIH1 overexpression (ARIH1 ORF). The lower panel depicts acetylated tubulin levels in scrambled control and ARIH1 knockdown cells treated with 10 nM paclitaxel for 3 and 6 h. HSP90 served as a loading control. Scram control is stable monoclonal control SUM159, generated by viral transduction of SUM159 cells with scrambled control shRNA construct. ARIH1 KD1 and KD2 are two different stable ARIH1 knockdown monoclones, generated by viral transduction of SUM159 by ARIH1 targeting shRNA construct. WT is wild-type PY8119 cells, generated by electroporating Cas9 enzyme alone into PY8119 cells. ARIH1 KO is ARIH1 knockout PY8119 cells, generated by electroporating the ARIH1 targeting CRISPR Cas9 system into PY8119 cells. Scale bar of 20 µM was used for immunofluorescence analysis.

MAP4 is a microtubule-associated protein that has been well-documented to play a pivotal role in promoting microtubule stabilization through tubulin acetylation and reducing microtubule depolymerization, processes essential for maintaining microtubule architecture and dynamics. Given the observed increase in MAP4 levels upon ARIH1 depletion, we hypothesized that ARIH1 loss induces microtubule stabilization through MAP4 upregulation. We assessed the impact of ARIH1 loss on microtubule stability and tubulin acetylation, a marker of stabilized microtubules. Immunofluorescence staining for beta-tubulin in SUM159 cells revealed distinct microtubule morphologies. Scrambled control (scram control) cells displayed a more diffuse beta-tubulin network, whereas ARIH1 knockdown (ARIH1 KD1 and KD2) cells exhibited a prominent spindle-like organization for the microtubules (Figure 3A). This shift toward a more stabilized microtubule architecture in ARIH1 KD cells suggests that ARIH1 negatively regulates microtubule stability.

Immunofluorescence analysis also revealed significantly higher levels of acetylated tubulin in ARIH1 KD1 and KD2 cells compared to the scram control (Figure 3B). Conversely, overexpression of ARIH1 (ARIH1 ORF) in SUM159 cells led to a marked reduction in acetylated tubulin levels compared to cells transfected with an empty vector (EV) (Figure 3C). These data confirm that ARIH1 directly or indirectly reduces microtubule stability.

Additionally, we validated the impact of ARIH1 on microtubule dynamics by assessing beta-tubulin organization in cells upon treatment with a microtubule-stabilizing agent such as paclitaxel. Several studies have demonstrated that the pre-treatment state of microtubules is a critical factor influencing the extent of paclitaxel-induced microtubule stabilization [9,10,11,12,13,14,15,16,17]. Given that ARIH1 loss is associated with microtubule stabilization, we anticipated that ARIH1-deficient cells would exhibit heightened responsiveness to the microtubule-stabilizing effects of paclitaxel. PY8119 WT cells treated with 100 nM paclitaxel for 24 h showed moderately spindled microtubules, while ARIH1 KO cells displayed highly bundled, stabilized microtubules under the same conditions (Figure 3D, left panel). Similarly, SUM159 cells treated with 10 nM paclitaxel for 5 h demonstrated pronounced spindle formation in ARIH1 KD1 and KD2 cells compared to the scram control (Figure 3D, right panel). These findings suggest that ARIH1 loss amplifies the stabilizing effect of paclitaxel on microtubules.

The enhanced microtubule stability in ARIH1 KD cells was further confirmed by evaluating tubulin acetylation after paclitaxel treatment. Immunofluorescence staining demonstrated significantly elevated levels of acetylated tubulin in ARIH1 KD1 and KD2 cells treated with 10 nM paclitaxel for 5 h compared to the scram controls under the same treatment conditions (Figure 3E). These data reinforce the hypothesis that ARIH1 loss enhances microtubule stabilization and augments the effect of microtubule-stabilizing agents like paclitaxel.

A Western blot analysis corroborated the immunofluorescence findings. In untreated SUM159 cells, acetylated tubulin levels were higher in ARIH1 KD1 and KD2 cells compared to the scram controls, while ARIH1 overexpression (ARIH1 ORF) led to a significant reduction in acetylated tubulin levels (Figure 3F, top panels). Upon treatment with 10 nM paclitaxel for 3 and 6 h, the acetylated tubulin levels further increased in ARIH1 KD1 and KD2 cells compared to the scram controls (Figure 3F, bottom panels). HSP90 was used as a loading control. These results demonstrate that ARIH1 loss promotes microtubule stabilization by increasing tubulin acetylation and reorganizing the microtubule architecture. This enhanced microtubule stability sensitizes ARIH1-deficient cells to the microtubule-stabilizing effects of paclitaxel.

### 3.4. ARIH1 Loss Improves Breast Cancer Cells’ Response to Paclitaxel

**Figure 4:**
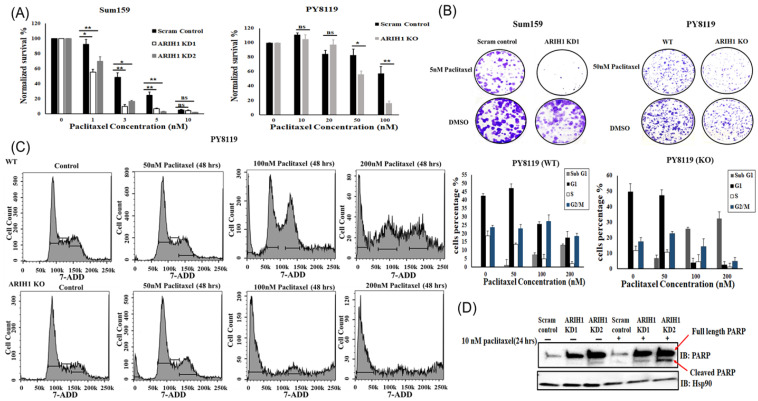
ARIH1 loss enhances breast cancer cell sensitivity to paclitaxel. (**A**) Dose–response curves of SUM159 (**left panel**) and PY8119 (**right panel**) cells treated with paclitaxel at increasing concentrations (1–10 nM for SUM159 and 10–100 nM for PY8119) for 96 h. (**B**) Representative images of colony formation assays in SUM159 cells treated with 5 nM paclitaxel or DMSO for 10 days and in PY8119 cells treated with 50 nM paclitaxel or DMSO for 10 days. (**C**) Flow cytometric analysis showing cell cycle distribution in PY8119 WT and ARIH1 KO cells treated with paclitaxel at 50 nM, 100 nM, and 200 nM for 48 h. (**D**) Western blot analysis showing full-length PARP and cleaved PARP in SUM159 cells treated with 10 nM paclitaxel for 24 h. Hsp90 was used as a loading control. Scram control is stable monoclonal control SUM159, generated by viral transduction of SUM159 cells with scrambled control shRNA construct. ARIH1 KD1 and KD2 are two different stable ARIH1 knockdown monoclones, generated by viral transduction of SUM159 by ARIH1 targeting shRNA construct. WT is wild-type PY8119 cells, generated by electroporating Cas9 enzyme alone into PY8119 cells. ARIH1 KO is ARIH1 knockout PY8119 cells, generated by electroporating the ARIH1 targeting CRISPR Cas9 system into PY8119 cells. Error bars represent the mean ± standard deviation from three independent experiments. * *p* < 0.05, ** *p* < 0.01, ns = not significant.

Given that paclitaxel exhibited a greater microtubule-stabilizing effect in ARIH1-deficient cells, we hypothesized that breast cancer cells lacking ARIH1 would demonstrate increased sensitivity and responsiveness to the cytotoxic effects of paclitaxel. We examined the effects of paclitaxel on cell viability using a dose–response assay (Figure 4A). SUM159 cells (Figure 4A; left panel) were treated with increasing concentrations of paclitaxel (1–10 nM) for 96 h. ARIH1 knockdown (KD1 and KD2) significantly reduced cell viability in a dose-dependent manner compared to scrambled controls. One nM paclitaxel reduced the cell viability of ARIH1 KD1 cells to 55.5% ± 3.5% and ARIH1 KD2 cells to 69.7% ± 6.1%, compared to 92.1% ± 6.2% in the scram control cells (*p* = 0.0228). Similarly, 3 nM paclitaxel further reduced the viability of ARIH1 KD1 cells to 10.01% ± 2.5% and ARIH1 KD2 cells to 16.8% ± 1.09%, compared to 48.5% ± 5.9% in the scram control cells (*p* = 0.0013). In addition, 5 nM paclitaxel reduced the viability of ARIH1 KD1 cells to 7.04% ± 0.7% and ARIH1 KD2 cells to 3.1% ± 0.57%, compared to 25.02% ± 4.3% in scram control cells (*p* = 0.0099), whereas at 10 nM, the effects converged due to paclitaxel’s saturation. The IC50 values revealed a marked increase in sensitivity in ARIH1 knockdown cells compared to the scrambled controls (scram control IC50 = 2.99 nM, ARIH1 KD1 IC50 = 1.05 nM, ARIH1 KD2 IC50 = 1.46 nM). ARIH1 KD1 cells exhibited a 2.85-fold increase in sensitivity, while ARIH1 KD2 cells showed a 2.05-fold increase compared to the scrambled controls.

In PY8119 cells (Figure 4A; right panel) treated with paclitaxel for 96 h, both WT and ARIH1 KO PY8119 cells displayed resistance and showed no significant difference in survival at 10 and 20 nM. However, 50 nM paclitaxel reduced the cell viability of ARIH1 KO PY8119 cells to 56.3% ± 4.5%, compared to 82.6% ± 8.5% in WT cells (*p* = 0.0213). Similarly, 100 nM paclitaxel further reduced the viability of ARIH1 KO PY8119 cells to 15.9% ± 2.9%, compared to 57.3% ± 9.7% in the WT cells (*p* = 0.0021), whereas at 150 nM, the effects converged due to paclitaxel’s saturation. In addition, ARIH1 KO cells showed significantly reduced IC50 values compared to the WT cells (WT IC50 = 168.89 nM, ARIH1 KO IC50 = 51.44 nM). ARIH1 KO cells were 3.28-fold more sensitive to paclitaxel than WT cells.

To assess the long-term proliferative potential of ARIH1 knockdown cells under paclitaxel treatment, a colony formation assay was performed (Figure 4B). In SUM159 cells, ARIH1 KD1 cells treated with 5 nM paclitaxel exhibited a significant reduction in colony formation compared to scrambled controls treated with the same concentration. This reduction was evident as a near absence of colonies in ARIH1 KD1 cells, indicating a profound loss of clonogenic capacity (Figure 4B; upper panel). In PY8119 cells, ARIH1 KO cells treated with 50 nM paclitaxel also showed a significant reduction in colony numbers compared to controls, further confirming the enhanced sensitivity of ARIH1-deficient cells to paclitaxel (Figure 4B: lower panel).

To investigate the mechanisms underlying the enhanced sensitivity of ARIH1-deficient cells to paclitaxel, we performed a flow cytometric analysis of the cell cycle (Figure 4C). In PY8119 WT cells treated with paclitaxel for 48 h, a dose-dependent increase in the sub-G1 population (indicative of apoptosis) was observed, along with G2/M arrest, which is consistent with paclitaxel’s mechanism of action. At 100 nM and 200 nM paclitaxel, sub-G1 populations reached 7.44% and 14.2%, respectively, in WT cells. In ARIH1 KO cells, the sub-G1 population increased significantly when compared to the WT cells at 100 nM (25.3%) and 200 nM (30.9%), indicating enhanced apoptosis upon ARIH1 loss.

We further confirmed the enhanced apoptotic response observed in ARIH1-depleted cells treated with paclitaxel by performing a Western blot analysis to detect PARP cleavage, a hallmark of apoptosis (Figure 4D). Sum159 cells were treated with 10 nM paclitaxel for 24 h, and both full-length PARP and cleaved PARP were evaluated. In the scramble control cells, paclitaxel treatment induced minimal PARP cleavage, indicating a limited apoptotic response. In contrast, ARIH1 knockdown cells (KD1 and KD2) displayed a marked increase in cleaved PARP levels compared to the scramble control. This suggests that ARIH1 depletion enhances paclitaxel-induced apoptosis.

These results demonstrate that ARIH1 loss sensitizes breast cancer cells to paclitaxel by reducing cell viability, impairing clonogenic potential, and enhancing apoptosis. In addition, it provides strong evidence that ARIH1 regulates MAP4, a key player in microtubule stabilization [9,10,11]. The loss of ARIH1 results in increased MAP4 expression and its association with beta-tubulin, leading to microtubule stabilization. This mechanism likely underpins the enhanced sensitivity of ARIH1-deficient cells to paclitaxel, as stabilized microtubules are more susceptible to microtubule-targeting agents [26,27,28,29]. This mechanism is consistent with previous reports demonstrating that MAP4 overexpression promotes microtubule stabilization [9,10,11,12,13,14], enhancing the response to paclitaxel and other microtubule-stabilizing agents in breast cancer and other cancers [15,16,17]. Collectively, our findings support ARIH1 as a critical regulator of microtubule dynamics and a promising therapeutic target for enhancing paclitaxel efficacy in breast cancer treatment.

## 4. Discussion

Our study uncovers a novel role for ARIH1 in modulating microtubule stability through its regulation of MAP4, a microtubule-associated protein known to enhance microtubule stabilization. This has significant implications for sensitizing cancer cells to microtubule-targeting agents like paclitaxel.

MAP4 is integral to microtubule dynamics, promoting stability by binding along microtubules and inhibiting depolymerization. An overexpression of MAP4 has been shown to increase microtubule stability, thereby enhancing the sensitivity of cancer cells to paclitaxel and other microtubule-stabilizing agents [15,16,17,26,27,28,29].

Paclitaxel exerts its anticancer effects by binding to β-tubulin, stabilizing microtubules through the inhibition of microtubule depolymerization, which ultimately disrupts mitotic spindle formation, halts cell division, and triggers apoptosis [30]. Since paclitaxel specifically interacts with preformed microtubules, the initial state of the microtubule network plays a crucial role in determining the extent of paclitaxel-induced stabilization and cytotoxicity. For instance, mutations that increase microtubule dynamic instability, even if they occur outside paclitaxel’s binding site, have been shown to drive resistance to the drug [7]. Conversely, microtubule stabilization has been linked to increased paclitaxel sensitivity, as demonstrated in ovarian cancer cells cultured on TGFBI-coated plates, which enhanced microtubule stability and strengthened paclitaxel’s cytotoxic effects [27]. Similarly, it has been shown that increasing the baseline stability of microtubules enhances both paclitaxel-induced microtubule stabilization and the drug’s ability to induce apoptosis [28].

Our findings align with these observations, demonstrating that ARIH1 loss leads to the upregulation of MAP4, resulting in increased microtubule stability and heightened sensitivity to paclitaxel. This suggests that ARIH1 may function as a negative regulator of MAP4, and its inhibition could be a viable strategy to enhance the efficacy of microtubule-targeting chemotherapies.

The role of ARIH1 in cancer chemoresistance has been increasingly recognized. As an E3 ubiquitin ligase, ARIH1 is involved in various cellular processes, including protein degradation and mitophagy. Recent studies have indicated that ARIH1-mediated mitophagy promotes therapeutic resistance in cancer cells [23]. For example, ARIH1 is widely expressed in cancer cells, notably in breast and lung adenocarcinomas, where its expression protects against chemotherapy-induced cell death.

Our study adds to this body of knowledge by elucidating a new mechanism through which ARIH1 contributes to chemoresistance—namely, by modulating microtubule stability via MAP4 regulation. By inhibiting ARIH1, we observed increased MAP4 levels, leading to microtubule stabilization and enhanced sensitivity to paclitaxel. This positions ARIH1 as a potential therapeutic target to overcome chemoresistance in breast cancer.

## 5. Conclusions

In conclusion, our research highlights the critical role of the ARIH1-MAP4 axis in regulating microtubule dynamics and influencing the response of cancer cells to microtubule-targeting agents. Targeting ARIH1 could represent a promising strategy for enhancing the efficacy of existing chemotherapeutic regimens, particularly in cases where resistance to microtubule-stabilizing agents poses a significant clinical challenge.

## Data Availability

The datasets used in this study are publicly available as noted in the text and Supplementary Materials.

## Supplementary Materials

The following supporting information can be downloaded at: https://www.mdpi.com/article/10.3390/cancers17050782/s1, File S1: The Original Western Blot figures.
