# Supplementary material for: ARIH1 Inhibition Promotes Microtubule Stability and Sensitizes Breast Cancer Cells to Microtubule-Stabilizing Agents
Source: Cancers (Basel). 2025 Feb 25;17(5):782. doi: 10.3390/cancers17050782 (PMC11898827; doi:10.3390/cancers17050782)

Figure 2

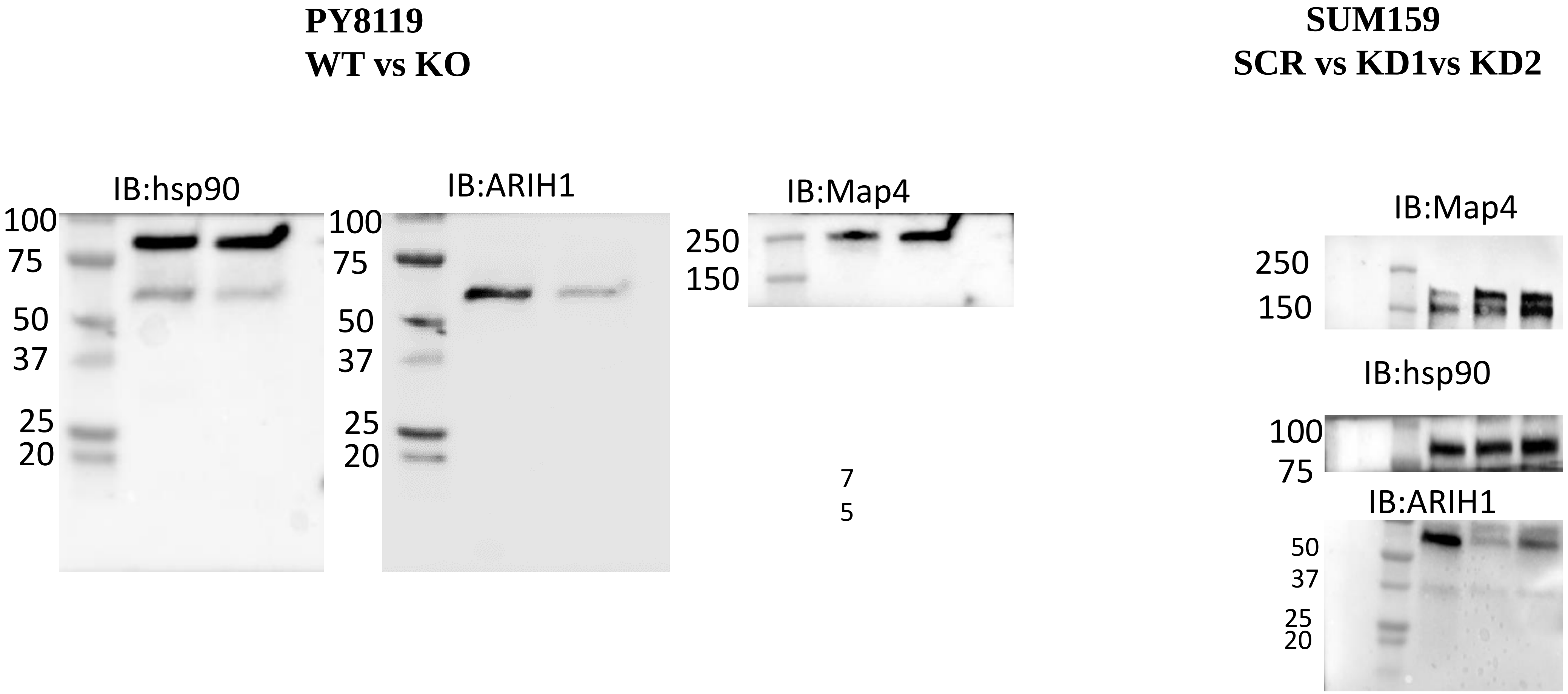
PY8119
WT vs KO
SUM159
SCR vs KD1vs KD2
IB:hsp90
100
75
50
37
25
20
IB:ARIH1
100
75
50
37
25
20
IB:Map4
250
150
7
5
IB:Map4
250
150
IB:hsp90
100
75
IB:ARIH1
50
37
25
20

Figure 3

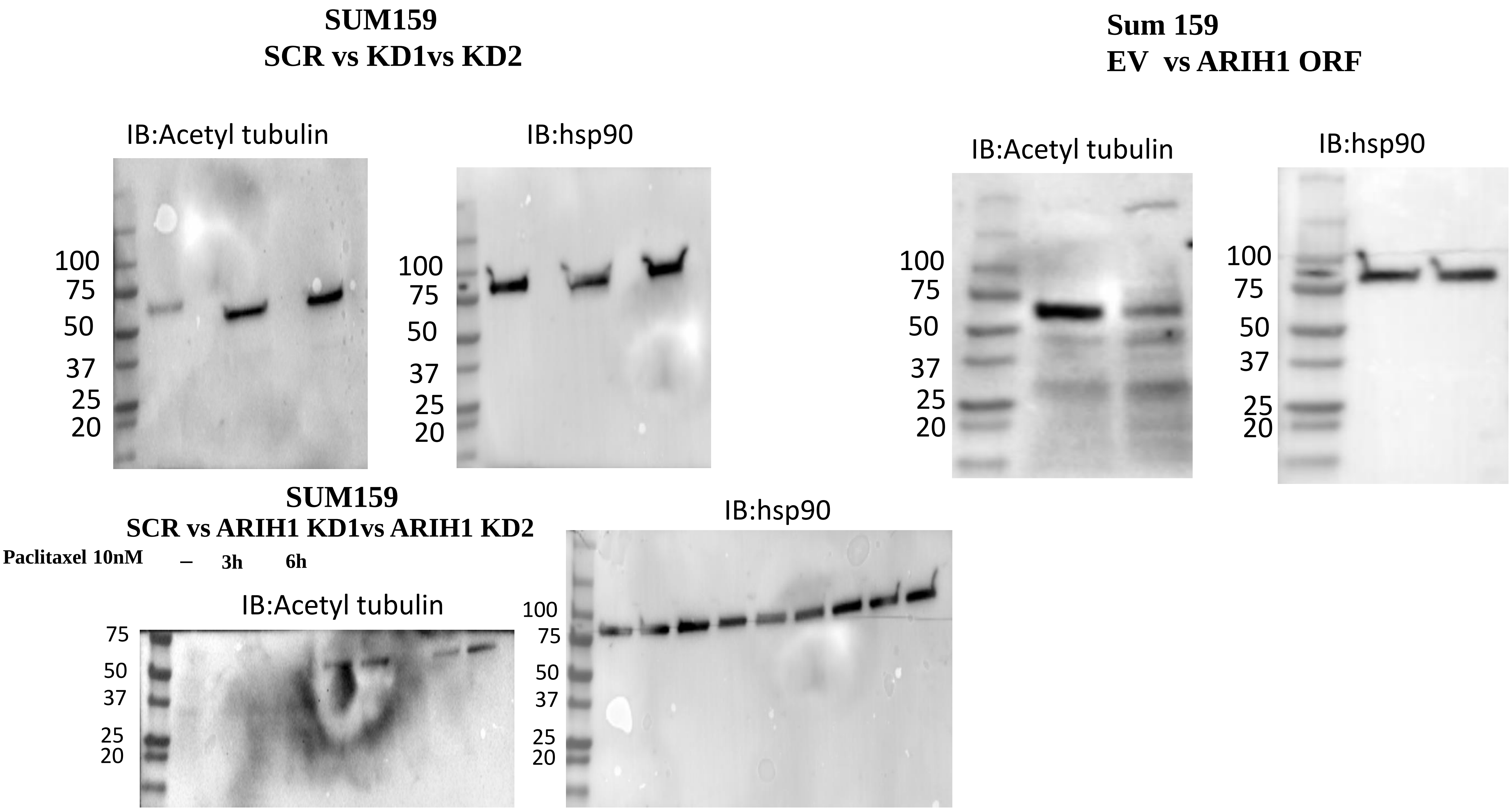
SUM159
SCR vs KD1vs KD2
IB:Acetyl tubulin
100
75
50
37
25
20
IB:hsp90
100
75
50
37
25
20
Sum 159
EV vs ARIH1 ORF
IB:Acetyl tubulin
100
75
50
37
25
20
IB:hsp90
100
75
50
37
25
20
SUM159
SCR vs ARIH1 KD1vs ARIH1 KD2
Paclitaxel 10nM
–
3h
6h
IB:Acetyl tubulin
75
50
37
25
20
IB:hsp90
100
75
50
37
25
20

Figure 4

SUM159
SCR vs ARIH1 KD1vs ARIH1 KD2

10 nM paclitaxel(24 hrs) +/-

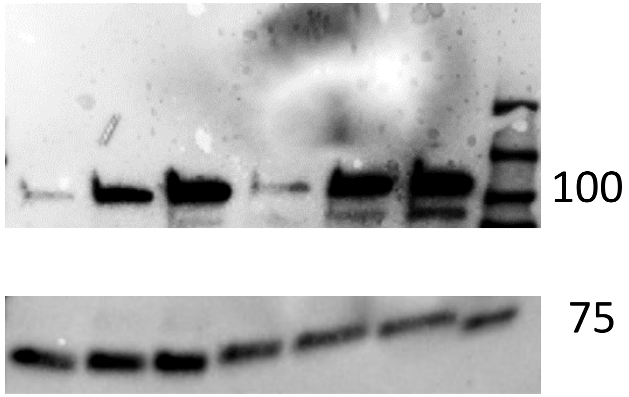

Supplement: Supplementary file 1 [file cancers-17-00782-s001.zip › cancers-3467279-supplementary.pdf]
